# Supplementary material for: Core Lab Adjudication of the ACURATE neo2 Hemodynamic Performance Using Computed-Tomography-Corrected Left Ventricular Outflow Tract Area
Source: J Clin Med. 2022 Oct 17;11(20):6103. doi: 10.3390/jcm11206103 (PMC9605387; doi:10.3390/jcm11206103)
Supplement: Supplementary file 1 [file jcm-11-06103-s001.zip › jcm-1935897-supplementary.pdf]

Table S1. Comparison among ACURATE*neo2* sizes and their interactions with methods (2D TTE & MDCT); The results of adjusted multiple Bayesian regression model.

Parameters	Variable	Beta	SE	95% Crl for Beta
EOA, (cm^2)	TTE/MSCT	-0.642	0.11	[-0.85, -0.43]
	Age (>82year/≤82 years)	-0.007	0.06	[-0.12, 0.11]
	Sex (Female/male)	-0.173	0.08	[-0.33, -0.02]
	BMI (≥30/ <30 kg/m^2)	0.318	0.18	[-0.04, 0.66]
	BSA, (1.6 m^2)	-0.16	0.36	[-0.89, 0.56]
	Neo2 size, 25mm/23mm	0.26	0.09	[0.068, 0.44]
	Neo2 size, 27mm/23mm	0.72	0.1	[0.51, 0.92]
	Size neo2 25mm* Group	0.436	0.14	[0.16, 0.7]
	Size neo2 27mm* Group	0.377	0.14	[0.11, 0.65]
EOAi, (cm^2/m^2)	TTE/MDCT	-0.214	0.04	[-0.29, -0.14]
	Age (>82year/≤82 years)	0.022	0.04	[-0.05, 0.1]
	Sex (Female/male)	-0.09	0.05	[-0.19, 0.01]
	BMI (≥30/ <30 kg/m^2)	0.063	0.06	[-0.04, 0.17]
	BSA, (1.6 m^2)	-0.054	0.13	[-0.31, 0.21]
	Neo2 size, 25mm/23mm	-0.092	0.06	[-0.21, 0.02]
	Neo2 size, 27mm/23mm	-0.054	0.07	[-0.18, 0.07]
	Size neo2 25mm* Group	0.084	0.05	[-0.01, 0.18]
	Size neo2 27mm* Group	0.021	0.05	[-0.08, 0.12]
SV, (ml)	TTE/MDCT	-7.296	3.77	[-14.45, -0.14]
	Age (>82year/≤82 years)	3.69	2.65	[-1.4, 8.79]
	Sex (Female/male)	-4.506	3.42	[-11.17, 2.25]
	BMI (≥30/ <30 kg/m2)	3.181	3.93	[-4.56, 10.73]
	BSA, (1.6 m^2)	7.126	8.44	[-9.49, 23.99]
	Neo2 size, 25mm/23mm	-8.781	3.98	[-16.29, -0.85]
	Neo2 size, 27mm/23mm	-6.408	4.32	[-15.02, 1.81]
	Size neo2 25mm* Group	4.997	2.85	[-0.61, 10.45]
	Size neo2 27mm* Group	1.389	2.8	[-4.02, 7.04]
SVi, (ml/m^2)	TTE/MDCT	-6.219	1.49	[-9.04, -3.3]
	Age (>82year/≤82 years)	2.619	1.43	[-0.27, 5.43]
	Sex (Female/male)	-3.28	1.89	[-6.86, 0.44]
	BMI (>30/ ≤30 kg/m2)	0.975	4.45	[-7.4, 9.84]
	BSA, (1.6 m^2)	7.369	9.12	[-10.5, 25.16]
	Neo2 size, 25mm/23mm	3.972	18.27	[-30.44, 41.81]
	Neo2 size, 27mm/23mm	11.533	18.06	[-24.37, 47.62]
	Size neo2 25mm* Group	3.004	1.83	[-0.5, 6.57]
	Size neo2 27mm* Group	-0.281	1.9	[-4.03, 3.37]

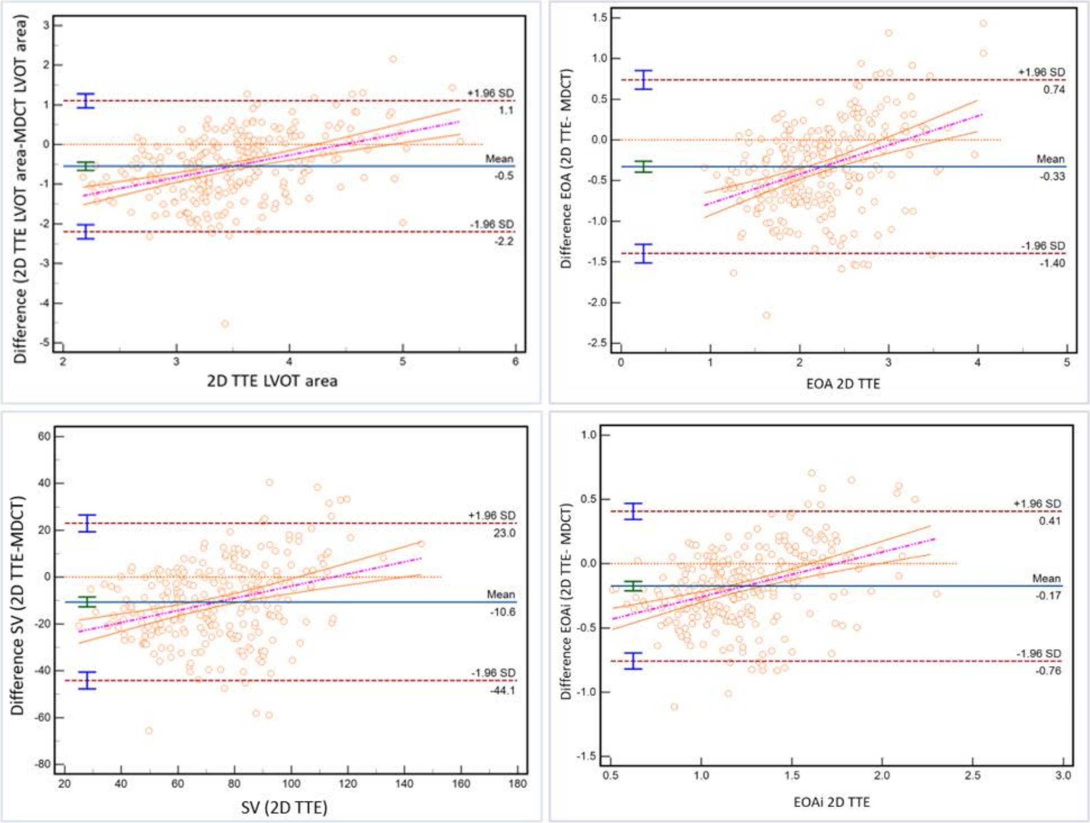

Figure S1: Bland-Altman plot showing the difference in calculated LVOT area (Upper left) EOA (Upper right), SV (Lower left) and EOAi (Lower right) using the 2D TTE and MDCT corrected method.

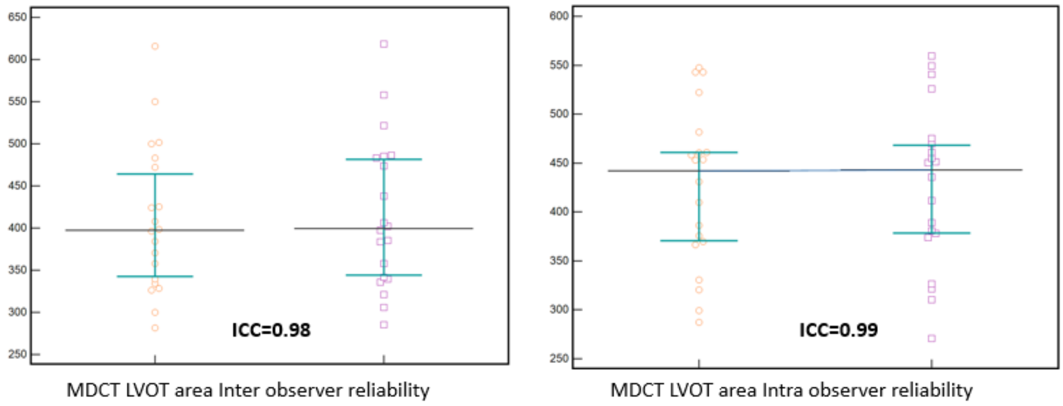

Figure S2: MDCT LVOT measurement; Inter observer (Left) and Intra observer reliability (Right) with excellent ICCs.

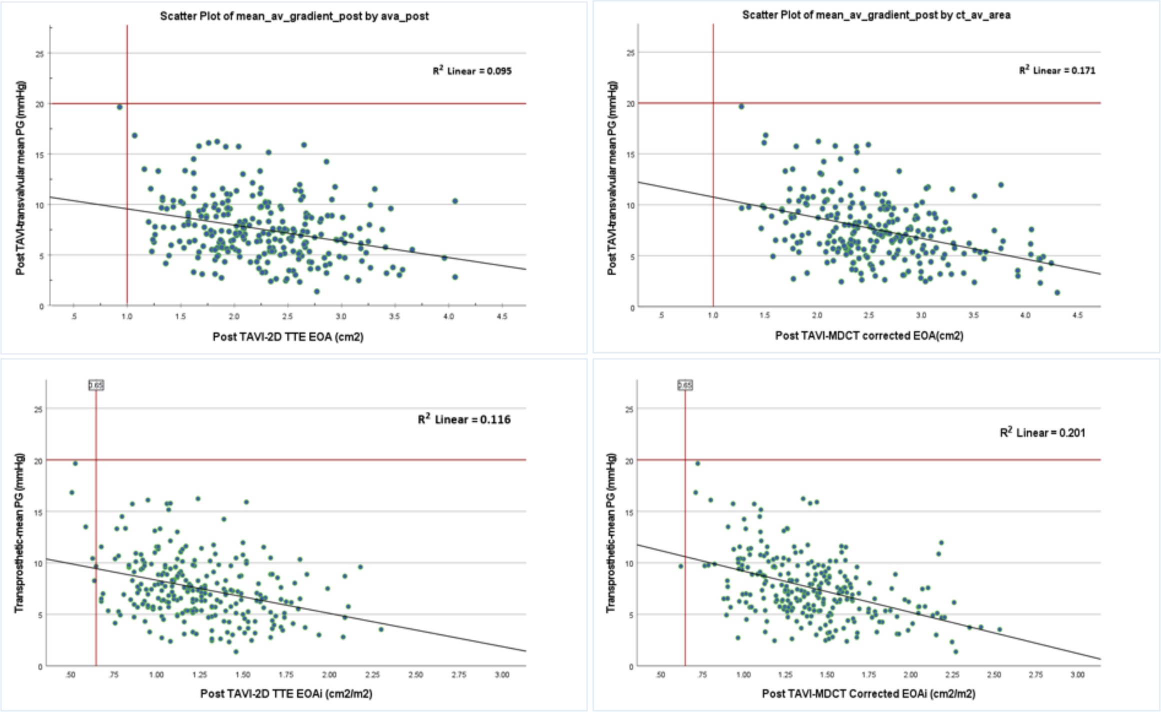

Figure S3: Scatter plots of the mean transvalvular gradient and EOA-2D TTE (Upper left), EOAi-2D TTE (Lower left), MDCT corrected EOA (upper right), MDCT corrected EOAi (Lower right) with a slightly better coefficient of determination (R^2) with the MDCT corrected parameters.

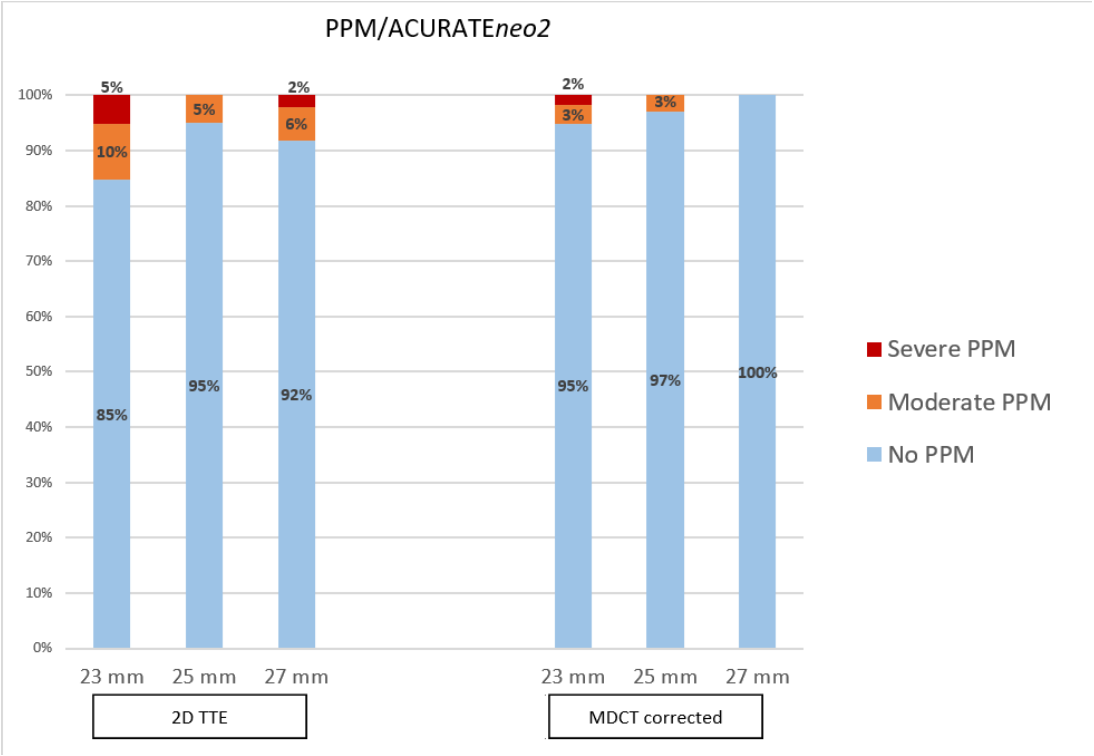

Figure S4: PPM (BMI adjusted) incidence, assessed by the 2D TTE and MDCT corrected methods and classified per ACURATE*neo2* size.

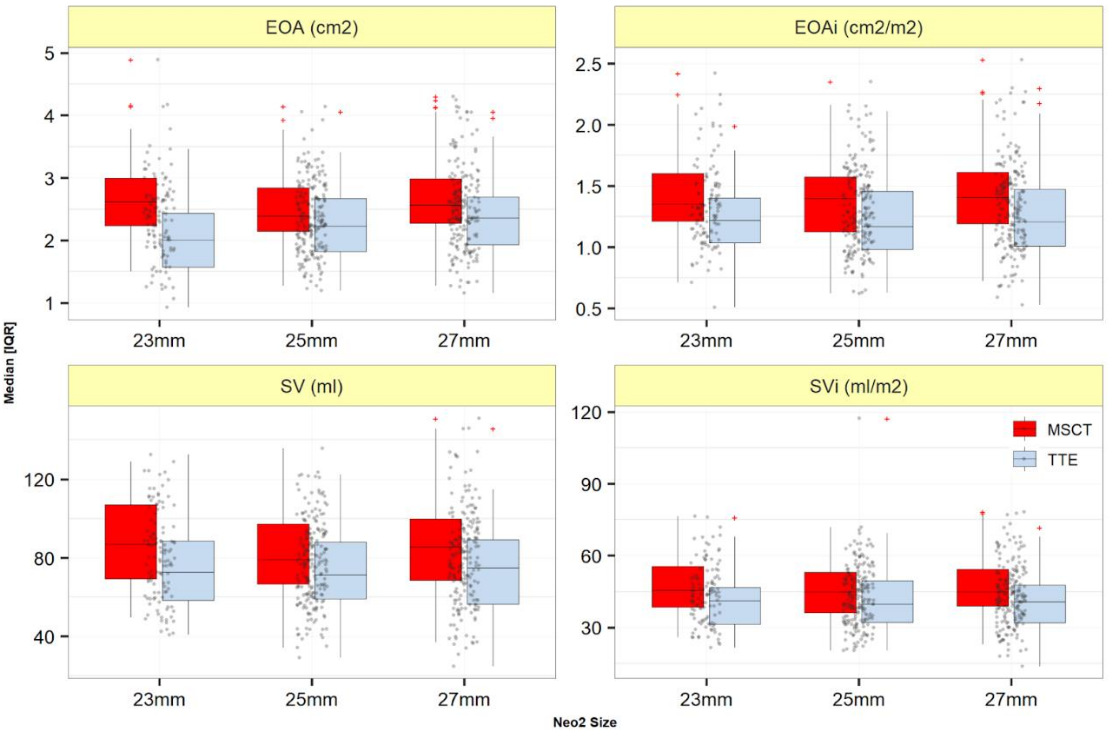

Figure S5: Inter valve size difference according to EOA, EOAi, SV and SVi per valve size.
